# Identifying Common Genes Related to Platelet and Immunity for Lung Adenocarcinoma Prognosis Prediction

**DOI:** 10.3389/fmolb.2020.563142

**Published:** 2020-10-29

**Authors:** Chengmao Zhou, Ying Wang, Lei Lei, Mu-Huo Ji, Jian-Jun Yang, Hongping Xia

**Affiliations:** ^1^School of Medicine, Southeast University, Nanjing, China; ^2^Department of Anesthesiology, Pain and Perioperative Medicine, The First Affiliated Hospital of Zhengzhou University, Zhengzhou, China; ^3^Department of Pathology, School of Basic Medical Sciences & Sir Run Run Hospital & State Key Laboratory of Reproductive Medicine & Key Laboratory of Antibody Technique of National Health Commission, Nanjing Medical University, Nanjing, China

**Keywords:** lung adenocarcinoma, immune, TCGA, platelet, nomogram

## Abstract

**Background:**

Although 1000s of immune-related and platelet receptor-related genes have been identified in lung adenocarcinoma, their role in prognosis prediction remains unclear.

**Methods:**

We downloaded mRNA data from the Cancer Genome Atlas Dataset (TCGA), and GSE68465 or GSE14814 data sets from the Gene Expression Omnibus (GEO) database.

**Results:**

The high-risk group’s overall survival (OS) time was lower than that of the low-risk group’s in TCGA (*p* = 1.15e-03). Additionally, the risk score was an independent prognostic survival factor for lung adenocarcinoma patients in TCGA (HR = 2.136, 95%CI = 1.553–2.937, *p* < 0.001). The model’s prognostic performance was verified with two independent GEO cohorts (GSE68465 and GSE14814). We also developed a nomogram and provided free webpage prediction tools.^1^ The mechanism of the high-risk group in this risk score may be have been related to somatic mutations and copy number changes. In addition, this risk score can distinguish the prognosis of the other two cancers (ACC, *p* < 0.001 and KIRP, *p* < 0.001). Also, among the other seven cancers, the OS prognosis for high and low risk groups show wide variation (*p* < 0.05).

**Conclusion:**

Our research demonstrates that CCNA2 and TGFB2 are potential diagnostic and prognostic biomarkers, as well as therapeutic targets in lung adenocarcinoma (LUAD). We also determined a novel and reliable prognostic score for lung adenocarcinoma prognosis. The online nomogram prediction tool that contains this risk score may also help clinical medical staff.

## Introduction

Lung cancer is one of the most common types of tumor (Siegel et al., 2019). Lung adenocarcinoma (LUAD) is one of the main subtypes of non-small cell lung cancer (NSCLC). In the United States, the 5-year survival rate for LUAD patients is as low as 20% (Lu et al., 2018). Most previous studies have used clinical baseline features (such as tumor size, cirrhosis, tumor number, and microvascular infiltration) and single-molecule biomarkers to construct prognostic models of lung adenocarcinoma. However, with the recent development of genome sequencing technology, the integration of prognostic-related gene signatures and traditional parameters has offered breakthroughs in predicting lung adenocarcinoma prognosis. However, these novel gene signatures need more utilization in clinical practice.

Recent studies have shown that platelets are central participants in systemic and local reactions to tumor growth (Haemmerle et al., 2018). Over 30% of patients with solid malignant tumors have thrombocytosis, which is a factor in diminishing patient survival. Tumor-derived interleukin 6 stimulates megakaryocyte production and thrombocytosis (Stone et al., 2012). Increasing platelet activity also promotes tumor growth and metastasis. Tumor cells induce platelet aggregation, leading to platelet tumor cell complexes. This reduces the monitoring of tumor cells by immunogenic cells and reduces the effects of blood turbulence and damage to tumor cells via shear stress in the blood flow (Goubran et al., 2013). Thrombocytosis can promote tumor cells’ metastatic potential in various ways. There are 21 platelet-related receptor genes that promote tumor development and expansion through non-inflammatory mechanisms (Menter et al., 2014). This includes stimulating MMP9 synthesis and generating adhesion molecules and growth factors (such as platelet-derived growth factor).

In addition, studies have revealed that platelets protect tumor cells from rendering immunotherapy useless (He et al., 2017). When platelets move around the circulatory system, they help tumor cells attach to the endothelium when they are trapped at a metastatic site, thereby achieving metastasis (Pavlovic et al., 2019). The unique biological characteristics of platelets enable them to participate in tumor cells’ immune response (Gaertner and Massberg, 2019).

Other studies have also related to the prediction model of survival for patients with lung cancer after chemotherapy or radiotherapy (Jochems et al., 2017). They also have used a lung adenocarcinoma prognosis model which uses 14 immune genes (Zhang et al., 2019). However, there is no prognosis model for lung adenocarcinoma using platelet and immunity. Therefore, this study explores the prognostic role of these platelet-related receptors and immune common genes in lung adenocarcinoma.

## Materials and Methods

### Data Collection

The mRNA expression and clinical data of patients were downloaded from the Cancer Genome Atlas Dataset (TCGA). The GSE68465 and GSE14814 data sets were downloaded from the Gene Expression Omnibus (GEO) database. Platelet-related receptor genes were obtained from the Gene Ontology Resource database.^2^ Immune-related receptor genes were obtained from the Immport database,^3^ and two common gene sets (platelet and immunity genes: PM genes) were populated. Copy number data and somatic mutation data were downloaded from TCGA. The tumor tissue data are pretreated by the following steps: (Siegel et al., 2019) deleting samples without clinical data; (Lu et al., 2018) deleting the sample data consisting of normal tissues; (Haemmerle et al., 2018) when the number of patients with a gene expression value of 0 exceeded 10%, the gene was also excluded; (Stone et al., 2012) keeping only the expression profiles of immune and platelet-related genes; (Goubran et al., 2013) patients with a survival time under 30 days were excluded; (Menter et al., 2014) the number of patients and specific clinical characteristics included in each data set can be seen in Supplementary XLS File 1.

The sample size is calculated on the website of http://powerandsamplesize.com/. Besides the TCGA data set as the training data set, we also verify it in two independent external data sets (GSE68465 and GSE14814).

### Identifying Genes Expressed Differential Genes Related to Platelet and Immunity (PMDEG) in TCGA-LUAD

Twenty-three annotated common genes related to platelets and immunity were used for differential expression analysis with the Limma version 3.5.1 R software package.

### Constructing Prognosis (PMDEG) Gene Signatures

Univariate and multiple Cox regression analyses were used to identify the metabolic genes involved in prognosis and to construct prognostic gene signatures. *p* < 0.01 in univariate Cox regression analysis was considered statistically significant. We used the R packages “survival” and “survminer” to explore the best cutoff point of the risk score and to draw a Kaplan–Meier survival curve. Then, we determined the optimal cutoff value for dividing patients into high- and low-risk groups with the “survminer” R software package’s “surv_cutpoint” function. Next, we studied the gene signatures’ prognostic values over time with the “survivalROC” R software package. A two-way log rank *p* < 0.05 was considered significant for survival analysis.

### Other Clinical Parameters’ Prognostic Gene Signatures’ Independence in TCGA

We conducted step-by-step univariate and multivariate Cox regression analysis.

### Constructing and Verifying Prediction Nomograms

Nomograms were established by incorporating all independent prognostic factors. We investigated nomogram calibration and identification (through the bootstrap method with 1,000 resamples) with the calibration chart and the consistency index (C-index).

### External Verification of Prognostic Gene Signatures and Genetic Changes

We calculated patients’ risk scores with genetic characteristics. We found that the construction and verification of the Kaplan–Meier analysis and the receiver operating characteristic (ROC) analysis were the same in the TCGA-LUAD cohort. The prognosis genes’ expression in gene signatures was verified at the mRNA level, and in the tumor immune estimation resource (TIMER) and protein-level databases. Then, we explored cBioportal cancer genomics to study the genetic changes in the gene signatures’ prognostic genes.

### Gene Set Enrichment Analysis

Gene set enrichment analysis (GSEA) identified enrichment terms in the TCGA’s PMDEG.

### Correlation Analysis

Unmatched *t*-test and routine one-way analysis of variance were performed to explore the relationship between score and clinical features. In the above analysis, *p* < 0.05 is considered to have statistical significance.

#### Meta-Analysis on the Difference of Tissue Expression and Survival Analysis of TGFB2 and CCNA2

We made a meta-analysis on the difference of tissue expression and survival analysis of TGFB2 and CCNA2 on the website of lung cancer explorer.^4^

#### TIMER Database Analysis

In the TIMER database,^5^ RNA-Seq expression profile data are used to detect immune cell infiltration. This includes neutrophils, macrophages, and dendritic cells in B cells, CD4+T cells, and CD8+T cells.

### Statistical Analysis

We used R v3.5.1 for statistical analysis and a Pearson χ^2^ test or Fisher’s exact test to explore qualitative variables. Unless specified above, *p* < 0.05 was considered statistically significant.

## Results

### Construction and Verification of Prognostic Metabolic Gene Signatures in TCGA

**FIGURE 1:**
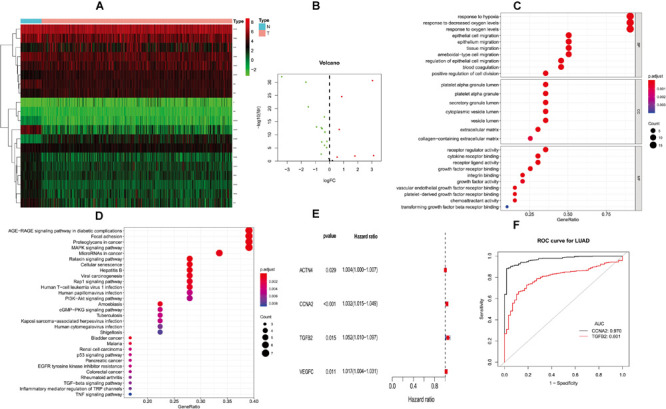
Differentially expressed PM-related genes and identifying hub HMGB-1-related genes. **(A)** Heat map of differentially expressed PM-related genes in the TCGA; **(B)** volcano plot of differentially expressed PM-related genes in the TCGA; **(C,D)** GO and KEGG for differentially expressed PM-related genes; **(E)** single-factor analysis for differentially expressed PM-related genes. **(F)** ROC results for PM-related genes in diagnosis of lung adenocarcinoma. PM, platelet and immunity; TCGA, the Cancer Genome Atlas Dataset; GO, gene ontology; ROC, receiver operating characteristic.

In order to construct the prognosis model of immune and platelet-related genes, it is necessary to screen out the prognosis genes, construct the prognosis model, and verify the efficacy externally. Twenty-three PM genes from TCGA-LUAD were included to train the prognostic models. We identified 20 PMDEGs (Figures 1A,B).

Gene ontology (GO) analysis showed that PMDEG-expressed genes were highly enriched in regulation of epithelial cell migration and response to hypoxia; KEGG pathway analysis showed that PMDEG-expressed genes were correlated with MAPK and p53 signaling pathway (Figures 1C,D).

The univariate Cox regression model found that four genes were correlated with survival, and then multivariate Cox analysis identified two genes for building the prognostic model (Figure 1E). The two genes included in this model were CCNA2 and TGFB2. In addition, we established a prognostic evaluation model based on risk score; its formula is risk score = (β *I* * Expi). In this formula, “*I*” represents the number of prognostic central genes, “Expi” represents the selected genes, and “βi” represents the weight of the genes, which is calculated based on multiple Cox regression. Risk score = CCNA2 × 0.0316489525026718 + TGFB2 × 0.0489706059459624. The patients were then divided into high-risk and low-risk groups according to the median risk score.

Next, we evaluated the diagnostic values of the CCNA2 and TGFB2 risk genes through ROC curve analysis of the lung cancer diagnoses. The CCNA2 and TGFB2 genes’ area under curve (AUC) values were 0.970 and 0.801, respectively (Figure 1F).

**FIGURE 2:**
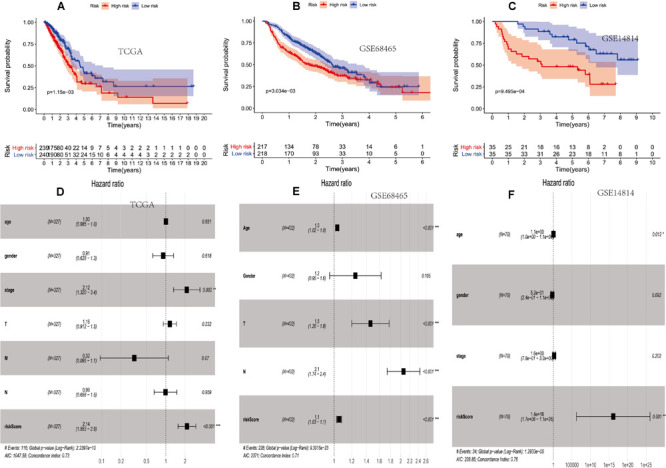
The prognostic value of two PM-related risk scores. **(A–C)** Kaplan–Meier curves for overall survival (OS) based on the risk score and single-factor and multi-factor analysis in the TCGA. **(D–F)** Kaplan–Meier curves for OS based on the risk score and multi-factor analysis in the GSE68465 and GSE14814 cohorts. PM, platelet and immunity; TCGA, Cancer Genome Atlas Dataset.

In order to prove that the score is a prognosis score, we conducted a survival analysis. The overall survival (OS) of the high-risk group was lower than that of the low-risk group in TCGA (*p* = 1.15e–03) (Figure 2A).

Then, the score was verified in the GSE68465 and GSE14814 cohorts. The OS of the high-risk group was lower than that of the low-risk group (*p* = 3.034e–03 and *p* = 9.495e–04) (Figures 2B,C).

It showed that clinical features and the prognosis model were independent prognostic factors of OS (Figures 2D–F).

**FIGURE 3:**
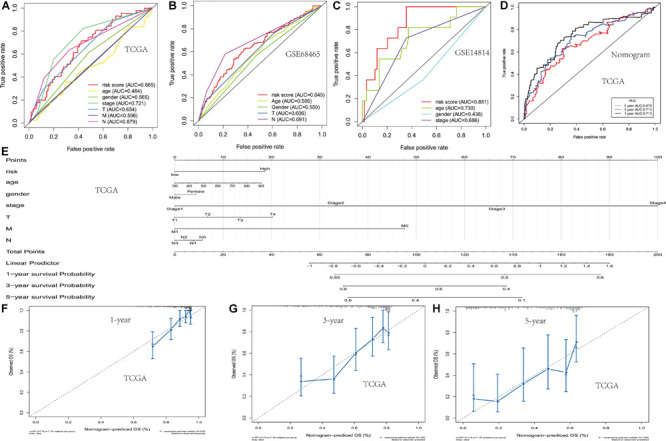
Development of the nomogram based on two PM-related genes and clinical features. **(A–C)** The receiver operating characteristic (ROC) curve of OS in TCGA and the GSE68465 and GSE14814 cohorts. **(D)** The ROC curve of the nomogram based on two PM-related genes in TCGA. **(E)** The nomogram based on two PM-related genes in TCGA. **(F–H)** 1-, 3-, and 5-year calibration curves of the nomogram in TCGA. PM, platelet and immunity; OS, overall survival; TCGA, Cancer Genome Atlas Dataset.

In order to establish and verify the performance of the prediction model composed of scores and clinical features, we verify the effectiveness of the model in three data sets. Whether in the training group or the verification group, the AUC value predicted by the risk score was similar to the AUC value predicted by the stage (Figures 3A–C). Nomograms were constructed by including tumor-node-metastasis (TNM) staging and prognostic models in TCGA. For the combined model, the AUCs of the 1-, 3-, and 5-year OS were 0.713, 0.713, and 0.678, respectively (Figures 3D,E). The calibration chart showed that nomograms performed best at predicting the 1-, 3-, and 5-year OS (Figures 3F–H). Moreover, we developed a nomogram using clinical features and risk score, and provided free personalized webpage prediction tools^1^ (Supplementary Figure 1).

The first step is to open the online tools website. Then, the txt format data are uploaded. The final step is to select patient characteristics and click on the calculation results. See Supplementary File Txt1 for the format of uploaded samples.

### Correlation Between the Risk Score and Immune Infiltrating Cellimmune, Checkpoint Member, Ubiquitination Gene, and Cell Cycle Gene

**FIGURE 4:**
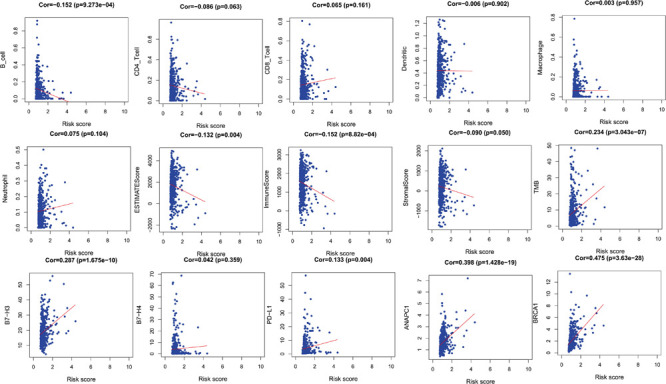
The relationships between two PM-related genes and other prognostic features (immune checkpoints, cell proliferation related genes, etc.). PM, platelet and immunity.

In order to verify the relationship between risk score and tumor-related prognostic factors such as immune factors, we analyzed the correlation between score and different tumor-related prognostic genes. In the TCGA-LUAD cohort, the risk score showed a high negative correlation with B cells (*r* = −0.152, *p* = 9.273e–04). The risk score showed a negative correlation with ESTIMATE score and immune score (*r* = −0.132, *p* = 0.004 and *r* = −0.152, *p* = 8.82e–04), but a positive correlation with tumor mutation burden (TMB) (*r* = 0.234, *p* = 3.043e–07). Moreover, the risk score showed a positive correlation with B7-H3 and PD-L1 (*r* = 0.287, *p* = 1.675e–10 and *r* = 0.133, *p* = 0.004). This indicated that patients with high risk scores may have benefitted from immunotherapy. The ubiquitination-related genes ANAPC1 and BRCA1 were also correlated with risk score (Figure 4).

### Risk Score’s Relationship With Copy Number Variation and Somatic Variation

**FIGURE 5:**
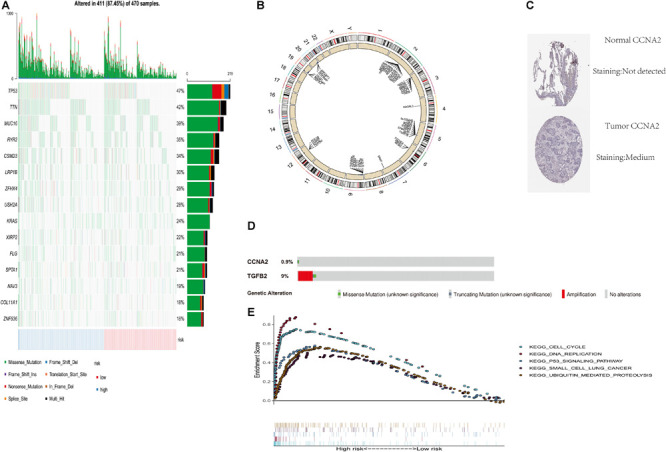
Genome-wide analysis of two PM-related genes risk scores (including mutation and copy number change). **(A)** Distribution of the first 15 mutant genes in high and low risk groups; **(B)** differential copy number in nine CLEC-2-related gene risk score high and low groups; **(C)** immunohistochemical results for risk score components in the HPA database; **(D)** mutation rate of TGFB2 and CCNA2 in the CBioportal database; **(E)** GSEA was performed among TCGA high-risk populations. PM, platelet and immunity; TCGA, Cancer Genome Atlas Dataset.

In order to verify the difference of copy number change and mutation between high and low score groups, we analyzed the copy number and mutation by using the data of TCGA database. TP53 and TTN had higher mutation frequency in tumors expressed in the high-risk group. There were also mutation genes with wide disparities between two risk groups (Figure 5A). Figure 5B shows the copy number differentials for the high- and low-risk scores, which were amplified in chromosomes 2, 4, 6, 7, 12, and 19, and lost in 3, 5, 9, 19, and 20. Immunohistochemical studies also showed that the expressions of CCNA2 in lung cancer and normal tissues were also different in the human protein atlas database (Figure 5C). The CBioportal database shows that TGFB2’s mutation rate among the lung cancer population was as high as 9% (Figure 5D).

### Gene Set Enrichment Analysis

In order to explore the related mechanism of different prognosis between high and low scores, we conducted GSEA analysis. Gene set enrichment analysis found five rich KEGG pathways among the TCGA-LUAD. Most enrichment pathways were related to lung cancer. Others were often dysregulated in cancer (such as DNA replication, cell cycle, P53 signaling pathway, and ubiquitination) (Figure 5E).

### Correlation Between the Prognosis Model and the Clinicopathological Characteristics

**FIGURE 6:**
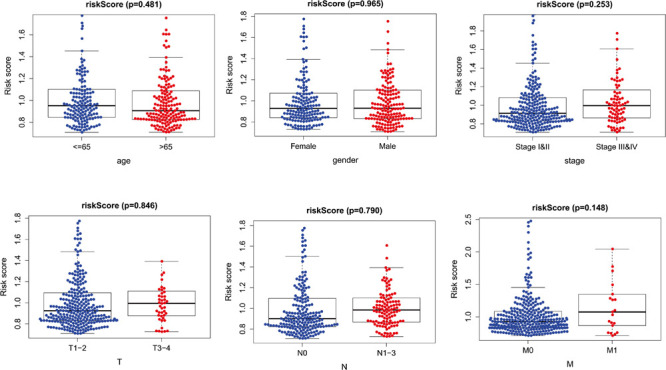
The relationships between two PM-related genes and clinical prognostic features. PM, platelet and immunity.

The risk score distribution among age, clinical stages, sex, and TNM was analyzed, as shown in Figure 6. Risk score was not associated with sex, age, tumor grade, or TNM stage (*p* > 0.05).

### Relationship Between Risk Score and Other Cancers

In addition, among the other seven cancers, the OS prognosis of the high and low risk groups for the risk score were different (*p* < 0.05) (Supplementary Figure 2).

### Meta-Analysis on the Difference of Tissue Expression and Survival Analysis of TGFB2 and CCNA2

We made a meta-analysis on the difference of tissue expression and survival analysis of TGFB2 and CCNA2 on the website of lung cancer explorer.^4^ The results are shown in Supplementary Figures 3, 4. The results show that there are statistical differences in the expression of these two genes in tumor tissues and normal tissues. However, only the survival analysis of CCNA2 has statistical differences.

## Discussion

Lung adenocarcinoma is a global public health challenge (Sivakumar et al., 2019), LUAD patients’ OS remains poor (Misono et al., 2019). And patients with the same pathological characteristics at diagnosis often have different prognosis (Wang et al., 2018). The genetic characteristics may facilitate the identification of new biomarkers for patients’ genetic profiles and clinical parameters. This study determined a new high-efficiency two-gene prognostic signature based on the TCGA data set and verified its effectiveness with the GSE68465 and GSE14814 data sets. Thus, our signature can stratify patients’ OS. In addition, the nomogram that included our signature performed best in predicting 1- and 3-year OS. Moreover, the free online web nomogram tool can help clinicians with precise treatments. GSEA indicates that the risk score is related to ubiquitination and cell cycle and that it is regulated by copy number variation and somatic mutation.

Many existing studies have revealed that the two genes used in the construction of this risk score are correlated with lung cancer prognosis. Studies have also shown that cyclin A2 and B1 expressions are prognostic biomarkers of lung cancer (Brcic et al., 2019). Additionally, it has also been reported that cyclin overexpression A2 is correlated with poor relapse-free survival (RFS) in a sample of 374 cases of lung cancer (*p* = 0.02) (Ko et al., 2013). Studies have also found that antisense oligonucleotides targeting TGF-β2 inhibits lung metastasis (Huber-Ruano et al., 2017). At the same time, the TGF-β2 antisense gene modifies allogeneic tumor vaccine belagenpumatucel-L and has an effect on advanced lung cancer (Nemunaitis et al., 2009). In addition, the TGF-β2 and TGF-β-R III signals transmitted by p38α/β regulate non-proliferative disseminated tumor cell dormancy and can regulate the microenvironment required for head and neck squamous cell carcinoma (HNSCC) metastasis (Bragado et al., 2013). And some studies have shown that single-nucleotide polymorphism (SNP) on CCNA2 promoter (rs769236) may be correlated with occurrence of colon, liver, and lung cancer (Kim et al., 2011). CCNA2 can promote the invasion and migration of NSCLC cells (Ruan et al., 2017). The functional polymorphism of TGFB1+869T > C may affect the sensitivity of NSCLC and the cellular microenvironment (Teixeira et al., 2011). TGFβ plays an important role in the invasion of tumor cells (Loomans and Andl, 2014). Transforming growth factor beta (TGFB) inducer homeobox 1 enhances the tumorigenesis of colorectal cancer by activating Wnt signal (Wang et al., 2017). And TGFB2 may play an important role in the connection between skin-mesenchymal transition (EMT) and TMB (Yang et al., 2020).

These two genes’ (TGFB2 and CCNA2) relationship with platelets and immunity has been reported by many tumor-related studies. α-Granule can be released after platelet activation, and it contains a growth factor (TGFB), which is associated with angiogenesis and the occurrence of malignant tumors (Golebiewska and Poole, 2015; Yuan and Liu, 2015; Yan and Jurasz, 2016). Studies show that platelet-derived TGFB promotes epithelial–mesenchymal transformation and transendothelial metastasis in tumor cells via TGFB/Smad and NF-kB pathways (Labelle et al., 2011). TGFB2 is a subtype of TGFB. Also, there are numerous studies indicating that TGFB2 is closely linked to immune responses. The antigen-presenting cells exposed by TGFB2 regulate thrombospondin-1 immunity (Mir et al., 2015). The novel tumor escape mechanism inhibits perforin expression in CD8+T cells mediated by TGFB2 (Hartana et al., 2018). Additionally, platelet-derived growth factor plays a key role in TGFB-mediated tumor progression (Gotzmann et al., 2006). In addition, platelet-derived growth factor can activate the surface receptor of vascular smooth muscle cells, thereby initiating the cell cycle and achieving the growth regulation of vascular smooth muscle cells (Bottger et al., 1988). Cyclin A2 is a subtype of cell cycle protein, and it is involved in DNA replication and the control of cell cycle checkpoint. Platelet-rich plasma upregulates CCNA2 expression by regulating Stat3 and p27, thereby increasing cell proliferation (Yu et al., 2015). And research shows that platelet-rich plasma releasate is closely related to CCNA2 (Tsai et al., 2017). Also, CCNA2 expression can reduce NO production by activating the immune response (Xu et al., 2016). Moreover, research reveals that CCNA2 may be a candidate target of immunotherapy for many cancer patients (Kondo et al., 2009).

Our research focuses on the prognostic role of platelet- and immune-related genes in lung adenocarcinoma. However, unlike previous studies, it is not limited to immune genes. Although our results have clinical significance, there are several limitations. First, our risk scores are based on retrospective data; prospective cohort studies are needed in different centers. Second, a more in-depth functional mechanism study of the risk score is needed.

## Conclusion

We have identified a novel two-gene prognosis signature related to platelet and immunity based on TCGA and GEO data sets. This signature may reflect the ubiquitinated and immune microenvironment disorder and provide potential biomarkers for ubiquitinated and immune targeted drug therapy and treatment response prediction. Moreover, we developed a nomogram using clinical features and risk score and provided free personalized webpage prediction tools.^1^

## Data Availability Statement

All datasets generated for this study are included in the article/Supplementary Material.

## Author Contributions

CZ, J-JY, and HX were major contributors in writing the manuscript. All authors analyzed the data.

## Conflict of Interest

The authors declare that the research was conducted in the absence of any commercial or financial relationships that could be construed as a potential conflict of interest.

## Footnotes

2 http://geneontology.org/

3 https://www.immport.org/home

4 http://lce.biohpc.swmed.edu/lungcancer/metagenename.php

5 https://cistrome.shinyapps.io/timer/

## Supplementary Material

Supplementary Figure 1 Operating Page of nomogram Online Web Page Prediction Tool Based on Risk Score and Clinical Characteristics. **(A)** Upload data operation page; **(B)** Patient Information Operation Interface Personalized Selection; **(C)** Forecast result interface.

Supplementary Figure 2 Correlation between Risk Score and other seven cancers.

Supplementary Figure 3 Meta-analysis on the difference of tissue expression of TGFB2 and CCNA2.

Supplementary Figure 4 Meta-analysis on the survival analysis of TGFB2 and CCNA2.
