# Supplementary material for: Identifying Common Genes Related to Platelet and Immunity for Lung Adenocarcinoma Prognosis Prediction
Source: Front Mol Biosci. 2020 Oct 29;7:563142. doi: 10.3389/fmolb.2020.563142 (PMC7658298; doi:10.3389/fmolb.2020.563142)
Supplement: Supplementary file 1 [file Data_Sheet_1.PDF]

age	gender	stage	T	M	N	riskScore	fustat
>65	Male	Stage I&II	T1-2	M0	N0	Low	1
>65	Female	Stage I&II	T1-2	M0	N1-3	Low	1
<=65	Male	Stage I&II	T1-2	M0	N0	Low	0
<=65	Female	Stage I&II	T1-2	M0	N0	Low	1
>65	Female	Stage I&II	T1-2	M0	N0	Low	0
>65	Female	Stage I&II	T1-2	M0	N0	Low	0
<=65	Male	Stage I&II	T1-2	M0	N0	Low	0
<=65	Female	Stage III&IV	T3-4	M0	N0	Low	0
<=65	Male	Stage I&II	T1-2	M0	N0	Low	0
>65	Male	Stage I&II	T1-2	M0	N0	Low	0
>65	Male	Stage I&II	T1-2	M0	N0	Low	0
<=65	Male	Stage III&IV	T1-2	M1	N0	Low	0
<=65	Female	Stage I&II	T1-2	M0	N0	Low	1
<=65	Female	Stage I&II	T1-2	M0	N0	Low	0
<=65	Male	Stage III&IV	T1-2	M0	N1-3	Low	1
<=65	Male	Stage III&IV	T3-4	M1	N1-3	Low	1
<=65	Male	Stage I&II	T1-2	M0	N1-3	Low	0
>65	Male	Stage I&II	T1-2	M0	N0	Low	0
>65	Female	Stage I&II	T1-2	M0	N0	Low	0
<=65	Female	Stage I&II	T1-2	M0	N1-3	Low	0
<=65	Female	Stage I&II	T1-2	M0	N0	Low	0
>65	Male	Stage III&IV	T1-2	M1	N0	Low	0
>65	Male	Stage III&IV	T1-2	M0	N1-3	Low	1
>65	Female	Stage I&II	T3-4	M0	N0	Low	0
>65	Female	Stage I&II	T1-2	M0	N0	Low	1
>65	Male	Stage I&II	T1-2	M0	N0	Low	0
>65	Female	Stage I&II	T1-2	M0	N0	Low	0
>65	Male	Stage I&II	T1-2	M0	N0	Low	0
>65	Male	Stage III&IV	T3-4	M0	N0	Low	0
<=65	Male	Stage I&II	T1-2	M0	N0	Low	1
>65	Male	Stage III&IV	T1-2	M1	N1-3	Low	1
<=65	Male	Stage I&II	T1-2	M0	N0	Low	0
>65	Female	Stage I&II	T1-2	M0	N0	Low	1
>65	Male	Stage I&II	T1-2	M0	N0	Low	1
>65	Male	Stage I&II	T1-2	M0	N0	Low	1
<=65	Female	Stage I&II	T1-2	M0	N0	Low	0
<=65	Female	Stage I&II	T1-2	M0	N1-3	Low	1
<=65	Male	Stage III&IV	T1-2	M1	N1-3	Low	1
>65	Female	Stage I&II	T1-2	M0	N0	Low	1
>65	Male	Stage I&II	T1-2	M0	N1-3	Low	1
>65	Male	Stage I&II	T1-2	M0	N1-3	Low	1
>65	Female	Stage I&II	T1-2	M0	N0	Low	0
>65	Male	Stage I&II	T1-2	M0	N0	Low	0
>65	Male	Stage I&II	T1-2	M0	N0	Low	0
>65	Female	Stage I&II	T1-2	M0	N1-3	Low	0
>65	Male	Stage I&II	T1-2	M0	N0	Low	0
>65	Female	Stage I&II	T1-2	M0	N0	Low	0
<=65	Female	Stage I&II	T1-2	M0	N0	Low	0

<=65	Male	Stage I&II	T1-2	M0	N0	Low	0
<=65	Female	Stage I&II	T1-2	M0	N0	Low	0
<=65	Female	Stage I&II	T1-2	M0	N0	Low	0
>65	Male	Stage I&II	T1-2	M0	N0	Low	0
<=65	Male	Stage I&II	T1-2	M0	N0	Low	1
<=65	Female	Stage I&II	T1-2	M0	N0	Low	0
>65	Female	Stage I&II	T1-2	M0	N0	Low	1
>65	Male	Stage III&IV	T1-2	M0	N1-3	Low	0
>65	Female	Stage III&IV	T1-2	M0	N1-3	Low	0
<=65	Female	Stage I&II	T3-4	M0	N0	Low	1
<=65	Female	Stage I&II	T1-2	M0	N1-3	Low	1
>65	Female	Stage I&II	T1-2	M0	N0	Low	0
<=65	Female	Stage I&II	T1-2	M0	N0	Low	0
<=65	Male	Stage I&II	T1-2	M0	N1-3	Low	0
>65	Male	Stage I&II	T1-2	M0	N0	Low	0
>65	Male	Stage III&IV	T1-2	M0	N1-3	Low	1
>65	Male	Stage I&II	T1-2	M0	N1-3	Low	0
>65	Female	Stage I&II	T1-2	M0	N0	Low	1
>65	Male	Stage I&II	T1-2	M0	N0	Low	0
<=65	Male	Stage I&II	T1-2	M0	N0	Low	1
<=65	Male	Stage I&II	T1-2	M0	N0	Low	1
>65	Female	Stage I&II	T1-2	M0	N0	Low	0
>65	Female	Stage I&II	T1-2	M0	N0	Low	0
<=65	Male	Stage I&II	T1-2	M0	N1-3	Low	0
<=65	Male	Stage I&II	T1-2	M0	N1-3	Low	0
>65	Male	Stage I&II	T1-2	M0	N0	Low	0
>65	Female	Stage I&II	T1-2	M0	N1-3	Low	1
>65	Female	Stage I&II	T1-2	M0	N0	Low	0
<=65	Male	Stage III&IV	T1-2	M0	N1-3	Low	1
<=65	Male	Stage I&II	T1-2	M0	N1-3	Low	0
>65	Male	Stage I&II	T1-2	M0	N0	Low	0
<=65	Female	Stage III&IV	T1-2	M0	N1-3	Low	1
>65	Male	Stage I&II	T1-2	M0	N0	Low	1
>65	Female	Stage I&II	T1-2	M0	N0	Low	0
>65	Male	Stage I&II	T1-2	M0	N0	Low	0
<=65	Female	Stage I&II	T1-2	M0	N0	Low	0
<=65	Male	Stage I&II	T1-2	M0	N1-3	Low	0
<=65	Female	Stage I&II	T1-2	M0	N1-3	Low	0
>65	Female	Stage I&II	T1-2	M0	N0	Low	1
>65	Female	Stage III&IV	T1-2	M0	N1-3	Low	1
>65	Female	Stage III&IV	T1-2	M0	N1-3	Low	0
>65	Male	Stage I&II	T1-2	M0	N0	Low	1
<=65	Female	Stage III&IV	T3-4	M0	N0	Low	0
<=65	Male	Stage I&II	T1-2	M0	N1-3	Low	0
<=65	Female	Stage I&II	T1-2	M0	N0	Low	0
<=65	Female	Stage I&II	T1-2	M0	N0	Low	1
<=65	Male	Stage I&II	T1-2	M0	N0	Low	0
<=65	Male	Stage III&IV	T3-4	M0	N1-3	Low	0
>65	Female	Stage I&II	T1-2	M0	N1-3	Low	1

<=65	Female	Stage III&IV	T1-2	M0	N1-3	Low	1
>65	Female	Stage I&II	T3-4	M0	N0	Low	0
<=65	Female	Stage I&II	T1-2	M0	N0	Low	0
<=65	Male	Stage I&II	T1-2	M0	N0	Low	0
<=65	Female	Stage I&II	T1-2	M0	N0	Low	0
<=65	Male	Stage I&II	T1-2	M0	N0	Low	0
>65	Female	Stage I&II	T1-2	M0	N0	Low	1
<=65	Male	Stage I&II	T1-2	M0	N0	Low	0
<=65	Male	Stage I&II	T1-2	M0	N1-3	Low	1
>65	Male	Stage I&II	T3-4	M0	N0	Low	1
>65	Female	Stage I&II	T1-2	M0	N1-3	Low	0
>65	Female	Stage I&II	T1-2	M0	N0	Low	1
<=65	Male	Stage I&II	T1-2	M0	N0	Low	0
>65	Male	Stage I&II	T1-2	M0	N0	Low	0
<=65	Male	Stage I&II	T1-2	M0	N0	Low	1
>65	Female	Stage I&II	T1-2	M0	N0	Low	1
>65	Male	Stage I&II	T1-2	M0	N1-3	Low	0
<=65	Female	Stage I&II	T1-2	M0	N0	Low	0
<=65	Female	Stage I&II	T1-2	M0	N1-3	Low	1
>65	Male	Stage III&IV	T1-2	M0	N1-3	Low	0
<=65	Male	Stage III&IV	T3-4	M0	N1-3	Low	1
<=65	Male	Stage III&IV	T1-2	M0	N1-3	Low	1
<=65	Male	Stage III&IV	T1-2	M0	N1-3	Low	1
<=65	Female	Stage I&II	T1-2	M0	N0	Low	0
>65	Male	Stage I&II	T1-2	M0	N0	Low	1
>65	Female	Stage I&II	T1-2	M0	N1-3	Low	0
>65	Female	Stage I&II	T1-2	M0	N0	Low	1
>65	Male	Stage I&II	T3-4	M0	N0	Low	0
>65	Male	Stage I&II	T1-2	M0	N0	Low	0
<=65	Male	Stage I&II	T1-2	M0	N0	Low	0
<=65	Female	Stage I&II	T1-2	M0	N0	Low	0
>65	Female	Stage III&IV	T1-2	M0	N1-3	Low	0
>65	Female	Stage I&II	T3-4	M0	N0	Low	0
<=65	Female	Stage I&II	T1-2	M0	N0	Low	0
<=65	Female	Stage I&II	T1-2	M0	N0	Low	1
<=65	Male	Stage I&II	T1-2	M0	N0	Low	0
<=65	Female	Stage I&II	T1-2	M0	N0	Low	0
>65	Male	Stage I&II	T1-2	M0	N0	Low	0
>65	Female	Stage III&IV	T1-2	M0	N1-3	Low	1
>65	Female	Stage I&II	T1-2	M0	N0	Low	0
>65	Female	Stage I&II	T1-2	M0	N0	Low	0
<=65	Female	Stage I&II	T1-2	M0	N0	Low	1
>65	Female	Stage III&IV	T1-2	M0	N1-3	Low	0
<=65	Female	Stage I&II	T1-2	M0	N0	Low	0
<=65	Male	Stage III&IV	T1-2	M1	N1-3	Low	0
>65	Female	Stage III&IV	T3-4	M0	N1-3	Low	0
<=65	Female	Stage I&II	T1-2	M0	N0	Low	0
>65	Female	Stage I&II	T1-2	M0	N0	Low	0
<=65	Male	Stage I&II	T1-2	M0	N1-3	Low	0

<=65	Female	Stage III&IV	T3-4	M0	N1-3	Low	1
<=65	Male	Stage I&II	T3-4	M0	N0	Low	0
>65	Female	Stage I&II	T1-2	M0	N0	Low	1
<=65	Female	Stage III&IV	T3-4	M1	N1-3	Low	1
>65	Male	Stage I&II	T1-2	M0	N0	Low	0
<=65	Female	Stage III&IV	T3-4	M0	N1-3	Low	1
<=65	Male	Stage I&II	T1-2	M0	N0	Low	1
<=65	Female	Stage I&II	T1-2	M0	N0	Low	0
>65	Female	Stage I&II	T1-2	M0	N1-3	Low	1
>65	Male	Stage I&II	T1-2	M0	N0	Low	0
>65	Female	Stage I&II	T1-2	M0	N0	Low	0
>65	Female	Stage III&IV	T1-2	M0	N1-3	Low	1
>65	Female	Stage I&II	T1-2	M0	N0	Low	0
<=65	Male	Stage III&IV	T1-2	M0	N1-3	Low	0
>65	Male	Stage I&II	T1-2	M0	N0	Low	1
>65	Male	Stage I&II	T1-2	M0	N0	Low	0
<=65	Female	Stage I&II	T1-2	M0	N1-3	Hight	0
<=65	Female	Stage III&IV	T1-2	M1	N0	Hight	0
>65	Female	Stage I&II	T1-2	M0	N0	Hight	0
>65	Female	Stage I&II	T1-2	M0	N0	Hight	0
<=65	Female	Stage I&II	T1-2	M0	N0	Hight	1
<=65	Female	Stage I&II	T1-2	M0	N1-3	Hight	0
>65	Male	Stage I&II	T1-2	M0	N0	Hight	1
<=65	Male	Stage I&II	T1-2	M0	N0	Hight	1
<=65	Male	Stage I&II	T1-2	M0	N0	Hight	0
<=65	Male	Stage I&II	T1-2	M0	N0	Hight	0
<=65	Female	Stage III&IV	T1-2	M0	N1-3	Hight	0
>65	Male	Stage III&IV	T1-2	M1	N0	Hight	0
<=65	Male	Stage III&IV	T3-4	M0	N1-3	Hight	0
<=65	Male	Stage I&II	T1-2	M0	N1-3	Hight	1
>65	Male	Stage I&II	T3-4	M0	N0	Hight	1
>65	Male	Stage III&IV	T1-2	M0	N1-3	Hight	0
<=65	Male	Stage III&IV	T1-2	M0	N1-3	Hight	0
<=65	Female	Stage III&IV	T1-2	M0	N1-3	Hight	0
<=65	Female	Stage I&II	T1-2	M0	N0	Hight	0
<=65	Female	Stage III&IV	T1-2	M0	N1-3	Hight	0
>65	Female	Stage I&II	T1-2	M0	N1-3	Hight	1
<=65	Male	Stage I&II	T1-2	M0	N0	Hight	0
<=65	Male	Stage I&II	T1-2	M0	N0	Hight	0
<=65	Male	Stage I&II	T1-2	M0	N1-3	Hight	1
>65	Male	Stage I&II	T1-2	M0	N1-3	Hight	0
<=65	Female	Stage I&II	T3-4	M0	N0	Hight	0
>65	Female	Stage I&II	T1-2	M0	N0	Hight	0
>65	Female	Stage I&II	T1-2	M0	N0	Hight	0
>65	Female	Stage I&II	T1-2	M0	N0	Hight	0
<=65	Male	Stage III&IV	T1-2	M1	N1-3	Hight	0
>65	Female	Stage I&II	T1-2	M0	N0	Hight	1
>65	Female	Stage I&II	T1-2	M0	N0	Hight	0
>65	Male	Stage III&IV	T1-2	M0	N1-3	Hight	0

>65	Female	Stage I&II	T1-2	M0	N0	Hight	1
>65	Male	Stage I&II	T1-2	M0	N0	Hight	0
<=65	Female	Stage III&IV	T1-2	M0	N1-3	Hight	0
>65	Female	Stage I&II	T1-2	M0	N0	Hight	0
>65	Female	Stage I&II	T1-2	M0	N0	Hight	1
<=65	Male	Stage I&II	T1-2	M0	N0	Hight	0
>65	Female	Stage III&IV	T3-4	M0	N0	Hight	0
>65	Female	Stage III&IV	T1-2	M0	N1-3	Hight	1
<=65	Female	Stage I&II	T1-2	M0	N0	Hight	0
<=65	Female	Stage III&IV	T1-2	M1	N0	Hight	0
>65	Male	Stage I&II	T1-2	M0	N1-3	Hight	1
>65	Female	Stage III&IV	T3-4	M0	N1-3	Hight	0
<=65	Female	Stage I&II	T1-2	M0	N0	Hight	0
<=65	Male	Stage III&IV	T3-4	M0	N1-3	Hight	0
>65	Female	Stage I&II	T1-2	M0	N0	Hight	0
<=65	Female	Stage I&II	T1-2	M0	N0	Hight	1
<=65	Female	Stage I&II	T1-2	M0	N1-3	Hight	0
>65	Male	Stage I&II	T1-2	M0	N0	Hight	0
>65	Male	Stage I&II	T1-2	M0	N0	Hight	1
>65	Female	Stage I&II	T1-2	M0	N1-3	Hight	0
<=65	Female	Stage I&II	T1-2	M0	N0	Hight	0
>65	Female	Stage I&II	T1-2	M0	N0	Hight	0
<=65	Female	Stage I&II	T1-2	M0	N0	Hight	0
>65	Female	Stage I&II	T1-2	M0	N0	Hight	0
<=65	Female	Stage I&II	T1-2	M0	N1-3	Hight	0
>65	Male	Stage III&IV	T3-4	M0	N1-3	Hight	1
<=65	Female	Stage I&II	T1-2	M0	N0	Hight	0
>65	Female	Stage I&II	T1-2	M0	N0	Hight	0
>65	Male	Stage I&II	T1-2	M0	N0	Hight	0
>65	Female	Stage III&IV	T1-2	M1	N0	Hight	0
<=65	Male	Stage I&II	T1-2	M0	N1-3	Hight	0
>65	Male	Stage I&II	T1-2	M0	N0	Hight	0
>65	Female	Stage I&II	T1-2	M0	N0	Hight	0
<=65	Male	Stage I&II	T1-2	M0	N0	Hight	0
<=65	Female	Stage I&II	T1-2	M0	N0	Hight	0
<=65	Male	Stage I&II	T1-2	M0	N1-3	Hight	0
<=65	Female	Stage I&II	T1-2	M0	N0	Hight	0
>65	Female	Stage I&II	T1-2	M0	N0	Hight	1
>65	Male	Stage III&IV	T3-4	M0	N1-3	Hight	0
>65	Male	Stage I&II	T1-2	M0	N0	Hight	0
>65	Male	Stage I&II	T1-2	M0	N1-3	Hight	0
>65	Male	Stage I&II	T1-2	M0	N1-3	Hight	1
<=65	Male	Stage I&II	T1-2	M0	N1-3	Hight	0
>65	Male	Stage I&II	T1-2	M0	N1-3	Hight	0
>65	Female	Stage I&II	T1-2	M0	N0	Hight	1
>65	Male	Stage I&II	T1-2	M0	N1-3	Hight	1
<=65	Female	Stage III&IV	T1-2	M0	N1-3	Hight	1
>65	Male	Stage I&II	T1-2	M0	N1-3	Hight	0
>65	Female	Stage I&II	T1-2	M0	N1-3	Hight	1

<=65	Male	Stage I&II	T1-2	M0	N0	Hight	0
>65	Female	Stage I&II	T1-2	M0	N0	Hight	0
>65	Female	Stage I&II	T1-2	M0	N0	Hight	0
<=65	Male	Stage I&II	T3-4	M0	N0	Hight	0
>65	Male	Stage III&IV	T3-4	M0	N1-3	Hight	0
>65	Male	Stage I&II	T1-2	M0	N1-3	Hight	0
>65	Male	Stage I&II	T1-2	M0	N1-3	Hight	0
<=65	Female	Stage I&II	T1-2	M0	N1-3	Hight	0
<=65	Male	Stage I&II	T1-2	M0	N0	Hight	1
<=65	Male	Stage I&II	T1-2	M0	N0	Hight	0
>65	Female	Stage I&II	T1-2	M0	N0	Hight	1
<=65	Female	Stage I&II	T1-2	M0	N0	Hight	1
>65	Male	Stage III&IV	T1-2	M0	N1-3	Hight	0
>65	Male	Stage III&IV	T1-2	M0	N1-3	Hight	0
>65	Female	Stage I&II	T1-2	M0	N0	Hight	0
<=65	Male	Stage I&II	T1-2	M0	N0	Hight	0
<=65	Female	Stage I&II	T1-2	M0	N1-3	Hight	0
<=65	Male	Stage I&II	T1-2	M0	N0	Hight	0
<=65	Male	Stage I&II	T1-2	M0	N0	Hight	1
<=65	Male	Stage I&II	T1-2	M0	N0	Hight	0
<=65	Female	Stage I&II	T1-2	M0	N0	Hight	1
>65	Male	Stage I&II	T1-2	M0	N0	Hight	0
<=65	Female	Stage III&IV	T1-2	M1	N0	Hight	0
>65	Female	Stage I&II	T1-2	M0	N0	Hight	0
>65	Male	Stage III&IV	T3-4	M0	N1-3	Hight	0
<=65	Male	Stage I&II	T1-2	M0	N0	Hight	1
<=65	Male	Stage III&IV	T3-4	M1	N1-3	Hight	1
<=65	Female	Stage III&IV	T3-4	M0	N1-3	Hight	0
<=65	Female	Stage III&IV	T1-2	M0	N1-3	Hight	1
<=65	Female	Stage I&II	T1-2	M0	N0	Hight	1
<=65	Female	Stage I&II	T1-2	M0	N0	Hight	1
<=65	Male	Stage I&II	T1-2	M0	N1-3	Hight	0
<=65	Female	Stage I&II	T1-2	M0	N0	Hight	1
<=65	Female	Stage I&II	T1-2	M0	N1-3	Hight	1
<=65	Female	Stage I&II	T1-2	M0	N0	Hight	1
>65	Male	Stage I&II	T3-4	M0	N0	Hight	1
<=65	Female	Stage I&II	T1-2	M0	N0	Hight	1
<=65	Male	Stage I&II	T1-2	M0	N1-3	Hight	0
<=65	Male	Stage I&II	T3-4	M0	N0	Hight	0
>65	Female	Stage I&II	T1-2	M0	N1-3	Hight	0
>65	Male	Stage I&II	T1-2	M0	N1-3	Hight	0
>65	Female	Stage I&II	T1-2	M0	N0	Hight	0
<=65	Male	Stage I&II	T1-2	M0	N0	Hight	0
<=65	Male	Stage I&II	T1-2	M0	N0	Hight	0
>65	Male	Stage I&II	T3-4	M0	N0	Hight	0
<=65	Male	Stage III&IV	T1-2	M1	N1-3	Hight	0
<=65	Male	Stage I&II	T1-2	M0	N1-3	Hight	0
<=65	Female	Stage III&IV	T3-4	M0	N1-3	Hight	0
>65	Female	Stage III&IV	T3-4	M1	N1-3	Hight	1

>65	Male	Stage I&II	T1-2	M0	N0	Hight	0
>65	Male	Stage III&IV	T1-2	M1	N0	Hight	1
>65	Male	Stage I&II	T1-2	M0	N0	Hight	0
>65	Male	Stage I&II	T1-2	M0	N0	Hight	0
>65	Male	Stage III&IV	T1-2	M0	N1-3	Hight	1
>65	Female	Stage III&IV	T1-2	M1	N0	Hight	1
>65	Male	Stage I&II	T1-2	M0	N0	Hight	0
>65	Male	Stage I&II	T1-2	M0	N1-3	Hight	0
<=65	Male	Stage I&II	T1-2	M0	N0	Hight	0
>65	Male	Stage I&II	T1-2	M0	N0	Hight	0
>65	Female	Stage III&IV	T1-2	M0	N1-3	Hight	1
<=65	Male	Stage I&II	T1-2	M0	N0	Hight	0
>65	Female	Stage I&II	T1-2	M0	N0	Hight	1
>65	Female	Stage III&IV	T1-2	M1	N0	Hight	0
>65	Male	Stage III&IV	T1-2	M0	N1-3	Hight	1
<=65	Male	Stage I&II	T1-2	M0	N1-3	Hight	1
>65	Female	Stage III&IV	T3-4	M0	N1-3	Hight	0
>65	Female	Stage III&IV	T3-4	M0	N1-3	Hight	0
<=65	Female	Stage I&II	T1-2	M0	N0	Hight	0
<=65	Male	Stage III&IV	T1-2	M0	N1-3	Hight	0
<=65	Male	Stage III&IV	T3-4	M0	N1-3	Hight	0
<=65	Female	Stage III&IV	T3-4	M1	N1-3	Hight	1
<=65	Female	Stage I&II	T1-2	M0	N0	Hight	0
>65	Female	Stage I&II	T1-2	M0	N0	Hight	1
<=65	Male	Stage III&IV	T1-2	M1	N0	Hight	0
<=65	Female	Stage I&II	T1-2	M0	N0	Hight	0
>65	Male	Stage I&II	T3-4	M0	N0	Hight	1
>65	Male	Stage III&IV	T3-4	M0	N1-3	Hight	1
>65	Female	Stage III&IV	T3-4	M0	N1-3	Hight	0
<=65	Male	Stage I&II	T1-2	M0	N0	Hight	0
>65	Female	Stage I&II	T1-2	M0	N0	Hight	0
<=65	Male	Stage I&II	T1-2	M0	N0	Hight	0
<=65	Male	Stage III&IV	T3-4	M0	N1-3	Hight	0
>65	Male	Stage III&IV	T3-4	M0	N0	Hight	1
