# Supplementary figures and images for: Identifying Common Genes Related to Platelet and Immunity for Lung Adenocarcinoma Prognosis Prediction
Source: Front Mol Biosci. 2020 Oct 29;7:563142. doi: 10.3389/fmolb.2020.563142 (PMC7658298; doi:10.3389/fmolb.2020.563142)

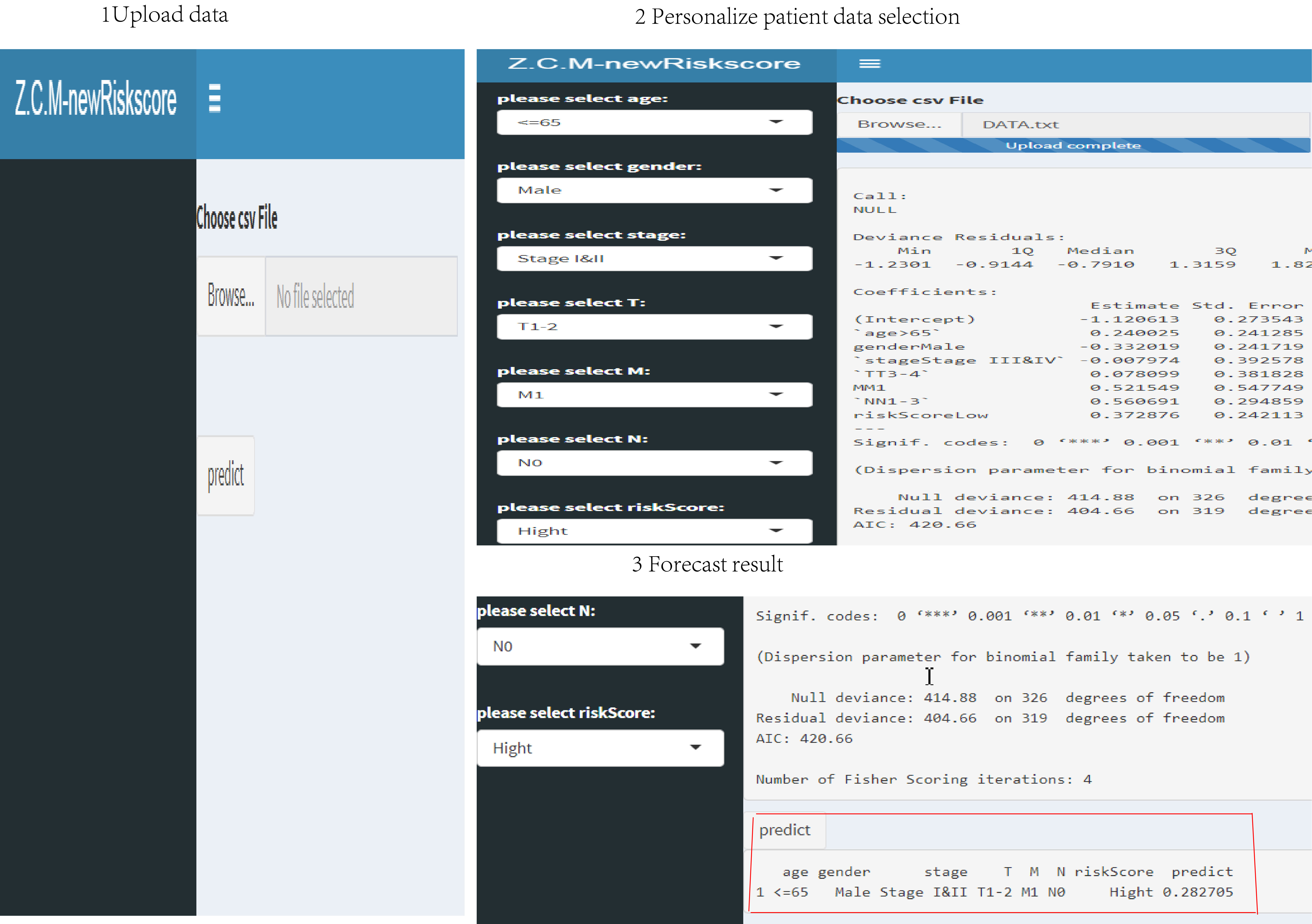

Supplement: Supplementary Figure 1 — Operating Page of nomogram Online Web Page Prediction Tool Based on Risk Score and Clinical Characteristics. (A) Upload data operation page; (B) Patient Information Operation Interface Personalized Selection; (C) Forecast result interface. [file Image_1.JPEG]

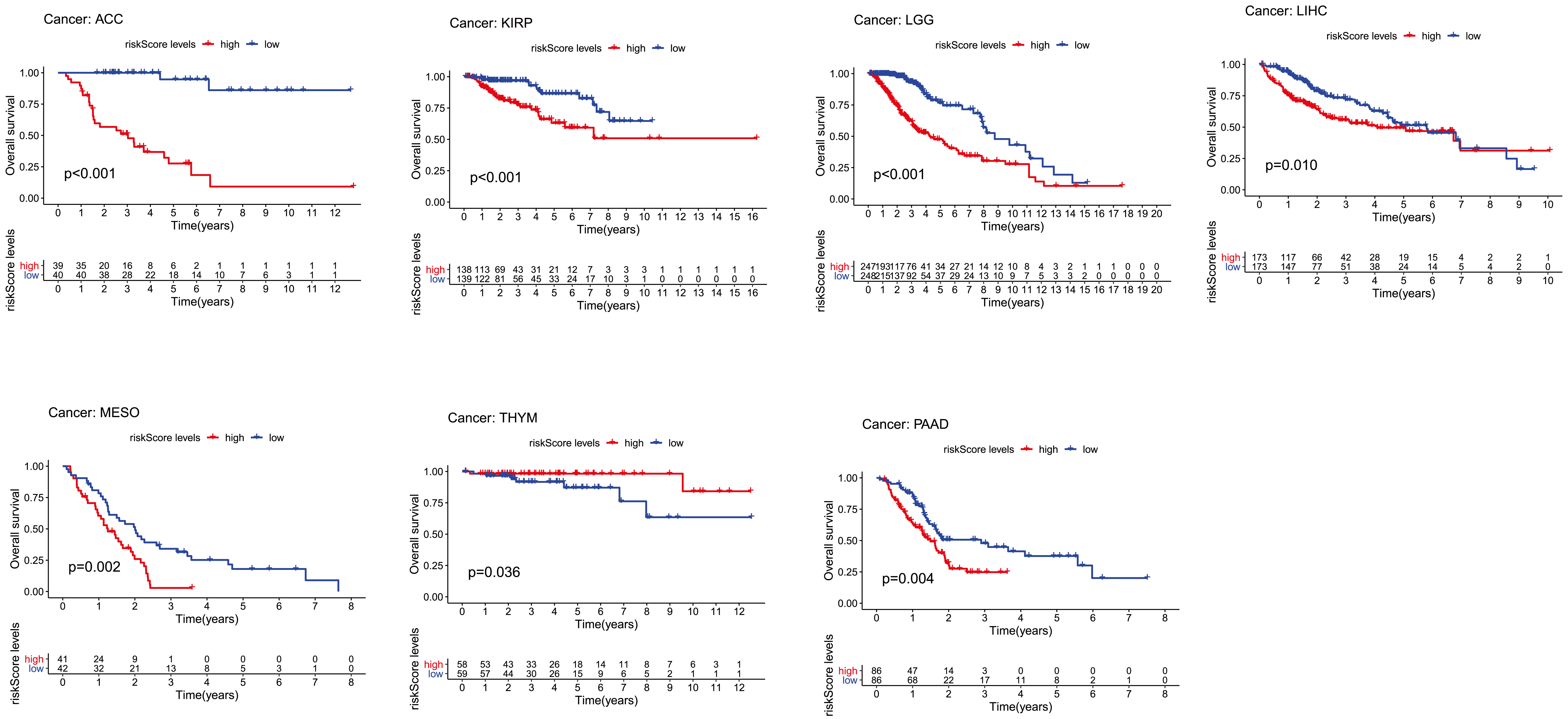

Supplement: Supplementary Figure 2 — Correlation between Risk Score and other seven cancers. [file Image_2.JPEG]

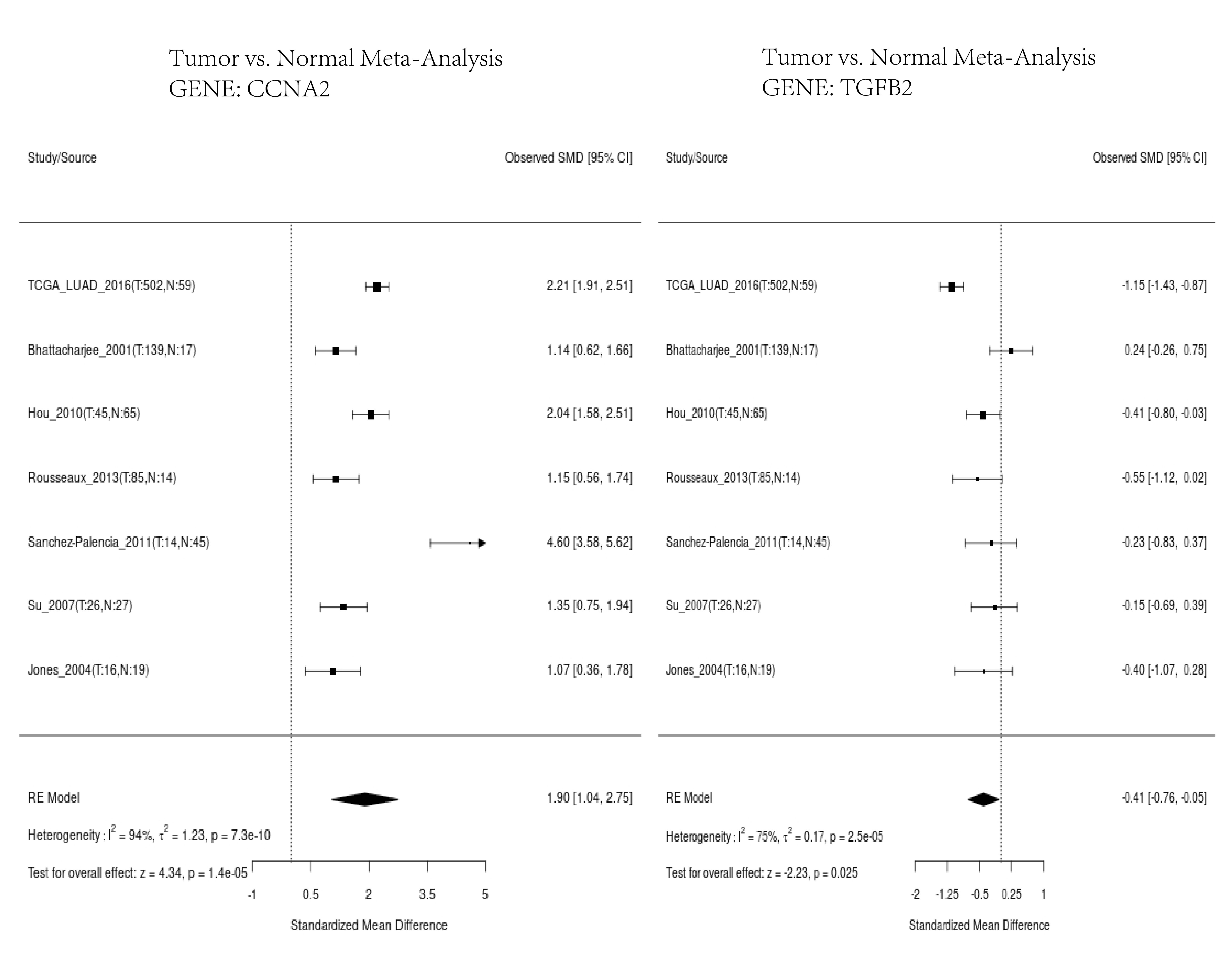

Supplement: Supplementary Figure 3 — Meta-analysis on the difference of tissue expression of TGFB2 and CCNA2. [file Image_3.JPEG]

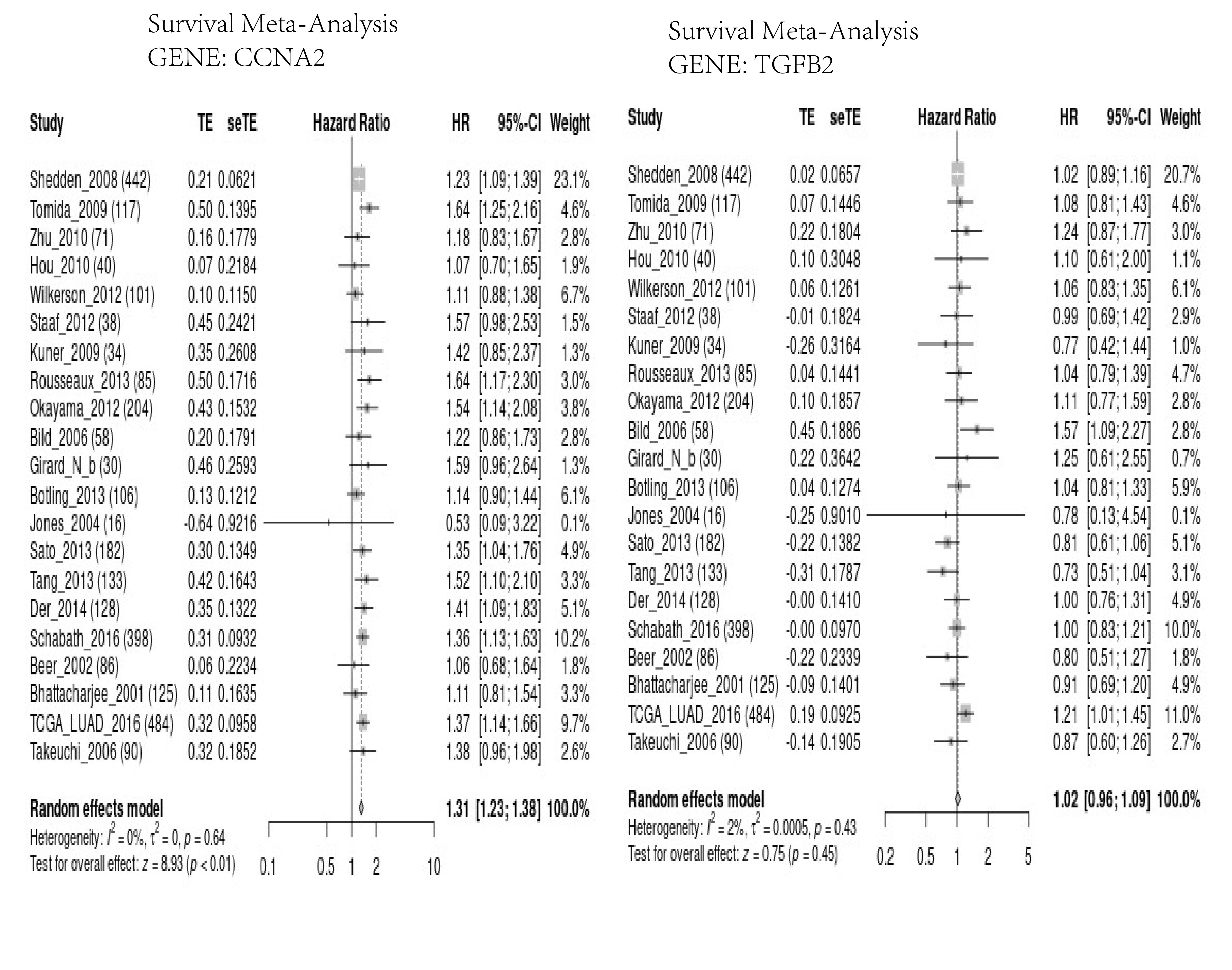

Supplement: Supplementary Figure 4 — Meta-analysis on the survival analysis of TGFB2 and CCNA2. [file Image_4.JPEG]
